# Conductive Iron Oxides Promote Methanogenic Acetate Degradation by Microbial Communities in a High-Temperature Petroleum Reservoir

**DOI:** 10.1264/jsme2.ME18140

**Published:** 2019-02-15

**Authors:** Souichiro Kato, Kaoru Wada, Wataru Kitagawa, Daisuke Mayumi, Masayuki Ikarashi, Teruo Sone, Kozo Asano, Yoichi Kamagata

**Affiliations:** 1 Division of Applied Bioscience, Graduate School of Agriculture, Hokkaido University Kita-9 Nishi-9, Kita-ku, Sapporo 060–8589 Japan; 2 Bioproduction Research Institute, National Institute of Advanced Industrial Science and Technology (AIST) 2–17–2–1 Tsukisamu-Higashi, Toyohira-ku, Sapporo 062–8517 Japan; 3 Institute for Geo-Resources and Environment, AIST 1–1–1 Higashi, Tsukuba 305–8567 Japan; 4 Technical Research Center, INPEX Corporation 9–23–20 Kitakarasuyama, Setagaya-ku Tokyo 157–0061 Japan; 5 Bioproduction Research Institute, AIST 1–1–1 Higashi, Tsukuba 305–8567 Japan

**Keywords:** methanogenesis, iron oxides, electric syntrophy, petroleum reservoir, microbial enhanced oil recovery

## Abstract

Supplementation with conductive magnetite particles promoted methanogenic acetate degradation by microbial communities enriched from the production water of a high-temperature petroleum reservoir. A microbial community analysis revealed that *Petrothermobacter* spp. (phylum *Deferribacteres*), known as thermophilic Fe(III) reducers, predominated in the magnetite-supplemented enrichment, whereas other types of Fe(III) reducers, such as *Thermincola* spp. and *Thermotoga* spp., were dominant under ferrihydrite-reducing conditions. These results suggest that magnetite induced interspecies electron transfer via electric currents through conductive particles between *Petrothermobacter* spp. and methanogens. This is the first evidence for possible electric syntrophy in high-temperature subsurface environments.

The methanogenic degradation of hydrocarbons in petroleum reservoirs has been attracting attention from the viewpoint of biogeochemistry and microbial-enhanced oil recovery (19). Acetate is an important intermediary metabolite of the methanogenic degradation of hydrocarbons and accumulates at concentrations up to 20 mM in petroleum reservoirs (24). The methanogenic degradation of acetate proceeds via the aceticlastic or syntrophic pathway. The aceticlastic pathway is mediated solely by aceticlastic methanogens, such as *Methanosaeta* spp. In the syntrophic pathway, syntrophic acetate-oxidizing bacteria oxidize acetate into H_2_ and CO_2_ (and/or formate), which are then utilized by hydrogenotrophic methanogens to produce CH_4_. The various environmental factors that influence which of the two pathways dominantly functions have been intensively investigated (6, 7, 15, 16).

Recent studies revealed that the syntrophic pathway may also be mediated by electric currents flowing through conductive solid materials instead of using H_2_ and/or formate as the electron carrier; this is specifically termed “electric syntrophy” or “direct interspecies electron transfer” (4). Microorganisms involved in electric syntrophy have the ability to exchange electrons with solid compounds, a process known as extracellular electron transfer (EET) (9). Electric syntrophy is mediated not only by naturally occurring conductive minerals, such as iron oxides and iron sulfides (4, 11), but also by artificial conductive materials, including graphite and activated carbon (12). These studies also revealed that methanogenesis via electric syntrophy is more efficient than that based on the diffusive transport of chemical compounds.

Although enhancements in the methanogenic degradation of acetate in the presence of conductive iron oxides has been demonstrated in various environments, such as those in rice paddy field soil (4) and thermophilic anaerobic digesters (27), it has not been investigated in subsurface environments, including high-temperature petroleum reservoirs. Considering the abundance of iron minerals in subsurface environments, methanogenesis dependent on electric syntrophy is expected to occur there. In the present study, microbial communities obtained from the production water of a high-temperature petroleum reservoir were cultivated in the presence or absence of conductive iron oxide (Fe_3_O_4_, magnetite) to investigate whether the methanogenic degradation of acetate is stimulated by the induction of electric syntrophy.

Production water and crude oil from a high-temperature petroleum reservoir, located in Yamagata Prefecture, Japan, were collected at the wellhead into gas-tight glass bottles flushed in advance with nitrogen gas. Ten milliliters of the production water was inoculated into vials (68-mL capacity) containing 10 mL of modified artificial seawater (MSW) medium. MSW medium comprised 18.7 mM NH_4_Cl, 2.2 mM KH_2_PO_4_, 15 mM MgCl_2_, 0.1 mM MgSO_4_, 0.5 mM CaCl_2_, 174.7 mM NaCl, 20 mM KHCO_3_, 40 mM 4-(2-hydroxyethyl)-1-piperazineethanesulfonic acid (HEPES), 20 mM sodium acetate, 0.005% (w/v) Bacto yeast extract, and 10 mL L^−1^ each of a trace element solution and vitamin solution (10). Magnetite and ferrihydrite were prepared as described previously (3) and supplemented to give a final concentration of 20 mM Fe. Bromoethane sulfonate (BES, final concentration 10 mM) was used as a specific inhibitor of methanogenic archaea. Cultures were incubated at 55°C under a N_2_:CO_2_ atmosphere (80:20 [v/v]) without shaking. The partial pressure of CH_4_ in the headspace was assessed using a gas chromatograph as described previously (6). When methanogenesis reached a plateau, 1 mL each of the enrichment cultures was subcultured to 20 mL of fresh medium. All culture experiments were conducted in triplicate and statistically analyzed using the Student’s *t*-test.

CH_4_ production rates and their lag times in the first generation of enrichment cultures were not significantly different between the presence and absence of magnetite (+Mag and Non-Fe, respectively) (Fig. 1A). In both enrichments, approx. 20 mmol L^−1^ of CH_4_ was produced from 20 mM acetate in *ca*. 40 d, whereas there was almost no CH_4_ produced (<0.1 mmol L^−1^) in the negative controls without acetate (data not shown). These results indicate that the acetate supplemented was completely converted to CH_4_ and CO_2_. Further sub-cultivations resulted in a reduction in lag times and increase in CH_4_ production rates, particularly in +Mag enrichments (Fig. 1B). In the fourth generation of subcultures, the maximum CH_4_ production rate in the +Mag enrichment (3.02±0.30 mmol L^−1^ d^−1^) was significantly higher than that in the Non-Fe enrichment (2.00±0.19 mmol L^−1^ d^−1^). These results clearly demonstrate that the presence of conductive iron oxides promotes the methanogenic degradation of acetate, and this appears to be due to the induction of electric syntrophy.

In order to identify the microorganisms involved in accelerated methanogenesis in the +Mag enrichment, we performed a clone library analysis targeting archaeal and bacterial 16S rRNA genes. We also analyzed another control enrichment culture supplemented with ferrihydrite (non-conductive and easily reducible iron oxides) and BES (specific inhibitor of methanogens), hereafter termed “+Fer+BES”, to discriminate between microorganisms involved in simple Fe(III) reduction and those involved in electric syntrophy. Total DNA was extracted from enrichment cultures using the FAST DNA Spin Kit for Soil (MP Biomedicals, Irvine, CA, USA) according to the manufacturer’s instructions. Partial 16S rRNA gene fragments were amplified by PCR with the primer pairs 27F and 907R for bacteria and A25F and A958R for archaea, as described previously (8). PCR products were purified using a QIAquick PCR Purification Kit (QIAGEN, Hilden, Germany), ligated into the pGEM-T Easy Vector (Promega, Madison, WI, USA), and cloned into *Escherichia coli* JM109 competent cells (Promega). The sequences of the cloned PCR products were elucidated at the Biomedical Center, Takara Bio (Kusatsu, Japan). A phylotype was defined as a unique clone or a group of clones with sequence similarity >97%. All phylotypes obtained in the present study are summarized in Tables S1 and S2.

The detection of only one archaeal phylotype (WD14, 100% identity to *Methanosaeta thermophila*) by a clone library analysis suggested that *Methanosaeta* spp. generated CH_4_ in the Non-Fe and +Mag enrichment cultures. *Methanosaeta* spp. were previously reported to have the ability to produce CH_4_ via electric syntrophy in addition to aceticlastic methanogenesis (20, 25). In contrast, bacterial community structures markedly differed in each enrichment culture (Fig. 2). The phylotype WD11 (phylum *Synergistetes*, 99% identity to *Anaerobaculum thermoterrenum*) dominated in all enrichment cultures, particularly in the Non-Fe enrichment. *Anaerobaculum* spp. have frequently been found in petroleum reservoirs (13) and are known as fermenting bacteria that utilize various sugars and amino acids (14). We assumed that *Anaerobaculum* spp. grew on the biomass produced by other microorganisms or on trace amounts of the yeast extract in enrichment cultures. Hence, the aceticlastic methanogens *Methanosaeta* spp. were considered to simply convert acetate to CH_4_ in the Non-Fe enrichment (Fig. 3A).

The phylotypes WD01 (class *Deltaproteobacteria*, 99% identity to *Desulfacinum subterraneum*), WD03 (phylum *Firmicutes*, 99% identity to *Thermincola ferriacetica*), and WD07 (phylum *Thermotogae*, 97% identity to *Thermotoga hypogea*) predominated under Fe(III)-reducing conditions (+Fer+BES). *Desulfacinum* spp. are frequently found in various geothermal environments, including petroleum reservoirs (13). Although the sulfate-reducing abilities of *Desulfacinum* spp. are well known, their Fe(III)-reducing abilities have not been tested (21). Considering the absence of sulfate in the medium and previous studies on the Fe(III)-reducing abilities of sulfate-reducing *Deltaproteobacteria* (26), *Desulfacinum* spp. appeared to reduce ferrihydrite in +Fer+BES enrichment cultures. *Thermincola* spp. are known as thermophilic Fe(III) reducers (2, 28), suggesting the contribution of this phylotype to ferrihydrite reduction in the +Fer+BES enrichment. *Thermotoga* spp. are frequently detected from petroleum reservoirs and generally known as thermophilic fermenters (13). The finding that some *Thermotoga* species, such as *T. maritima*, *T. subterranean*, and *T. lettinga*, have Fe(III)-reducing abilities (1, 22) suggests the involvement of this phylotype in ferrihydrite reduction.

The most dominant phylotype in the +Mag enrichment was WD13 (phylum *Deferribacteres*, 99% identity to *Petrothermobacter organivorans*), followed by WD06 (phylum *Thermotogae*, 99% identity to *Petrotoga sibirica*). *P. organivorans* was recently isolated from the same petroleum reservoir used in the present study as a novel genus strain in the phylum *Deferribacteres* (23). Similar to other strains of *Deferribacteres*, *P. organivorans* has the ability to utilize insoluble iron oxides as an electron acceptor (23). *Petrotoga* spp. have only been found in petroleum reservoirs (13) and are generally known as fermenting bacteria; their Fe(III)-reducing abilities have not yet been tested. However, the finding that most species utilize insoluble elemental sulfur as an electron acceptor (18) suggests EET abilities in *Petrotoga* spp.

It is important to note that completely different microorganisms expected to have EET abilities were dominant in the +Mag and +Fer+BES enrichments. Since methanogenesis was inhibited, the dominant bacteria in the +Fer+BES cultures are assumed to simply acquire energy through ferrihydrite reduction (Fig. 3B). In contrast, because magnetite is recalcitrant to microbial reduction (5) and syntrophic methanogenesis via electric currents may be mediated by magnetite particles (4), it is highly likely that the bacteria that dominated the +Mag enrichment (*i.e*., *Petrothermobacter* spp. and possibly *Petrotoga* spp.) acquired energy via electric syntrophy with methanogens as partners (Fig. 3C). To date, only two groups of bacteria (*i.e*., *Deltaproteobacteria* and *Firmicutes*) have been proven to play a role in methanogenic acetate degradation via electric syntrophy (4, 27). This study is the first to suggest the involvement of other microbial phyla (*Deferribacteres* and possibly *Thermotogae*) in electric syntrophy. In the +Mag enrichment, *Methanosaeta* was the only methanogenic archaea detected by the clone library analysis (Table S2). *Methanosaeta* spp. were previously reported to have the ability to produce CH_4_ via electric syntrophy (20, 25), and were assumed to acquire additional energy via electric syntrophy in addition to aceticlastic methanogenesis (Fig. 3C).

Pan *et al*. (17) recently reported the influence of iron oxide minerals on methanogenic acetate degradation by microbial communities derived from a petroleum reservoir. Although they demonstrated the promotion of methanogenesis by supplementation with magnetite or β-FeOOH (akaganeite) particles, they did not observe a clear change in the microbial community structure or induction of electric syntrophy. This may be due to the insufficient enrichment of microbial communities because they did not conduct any subculturing. Several generations of subculturing are often required to observe the effects of supplementation with conductive materials, particularly when the abundance of microorganisms involved in electric syntrophy is low in inoculum samples (11). Another possible reason is that the reducing agent (*ca*. 2 mM Na_2_S) added to the culture medium affected the conductivity of iron oxides through chemical reduction.

In conclusion, the present study demonstrated for the first time that conductive minerals promote the methanogenic degradation of acetate in high-temperature petroleum reservoirs, most likely due to the induction of electric syntrophy. The confirmation of methanogenesis via electric syntrophy in petroleum reservoirs provides insights for understanding biogeochemical processes and also the development of new strategies for microbial-enhanced oil recovery by promoting the conversion of residual hydrocarbons to CH_4_ *in situ*. Further investigations into the effects of iron oxide minerals in petroleum reservoirs and also experiments using co-cultures of *Petrothermobacter* spp. and *Methanosaeta* spp. will shed light on the importance of electric syntrophy in the degradation of hydrocarbons in subsurface environments.

## Nucleotide sequence accession numbers

The GenBank/EMBL/DDBJ accession numbers for the 16S rRNA gene sequences of clones from this study are LC378401–LC378414.

## Supplementary Information



## Figures and Tables

**Fig. 1:**
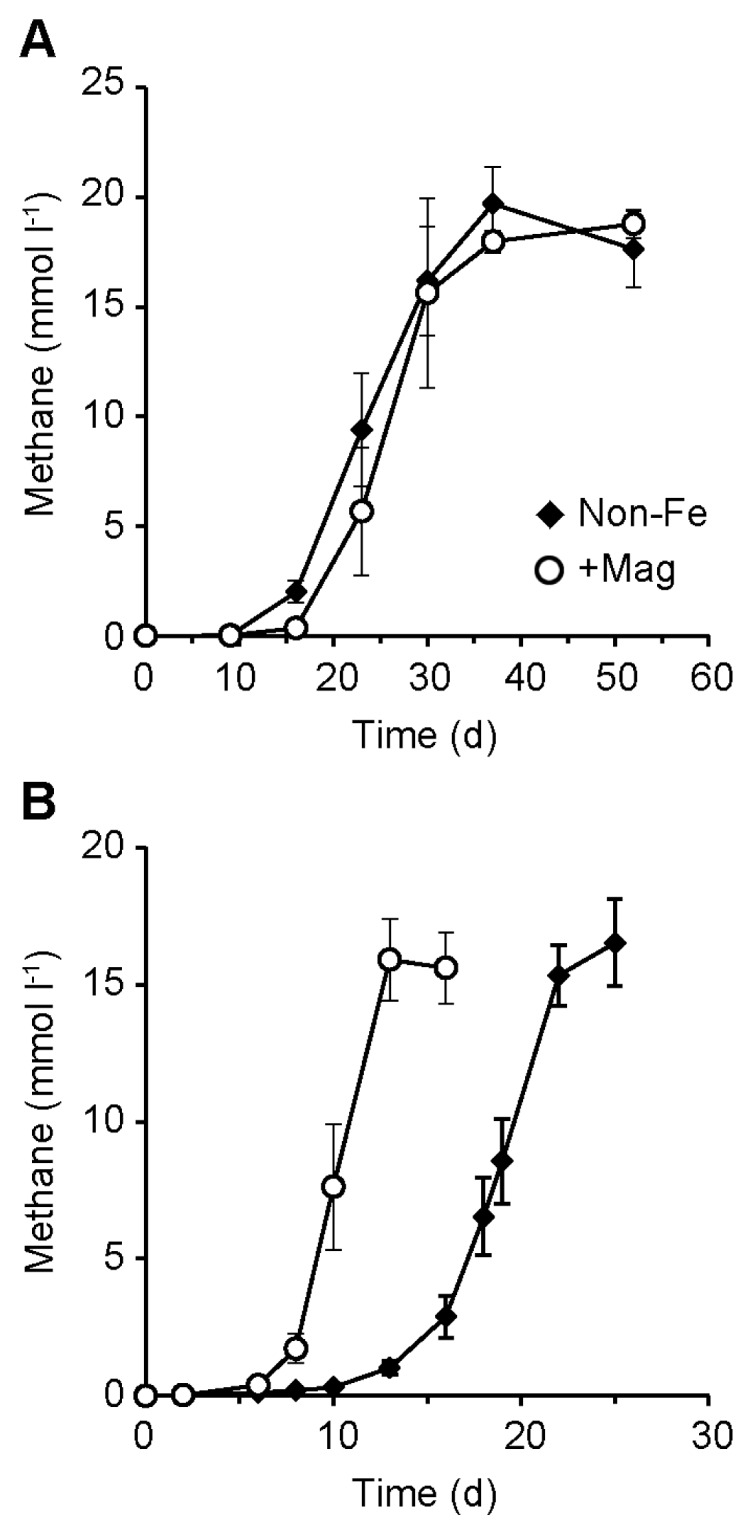
Methanogenesis by microbial communities derived from a petroleum reservoir. The results from first (A) and fourth (B) generation enrichment cultures in the presence (+Mag, open circles) or absence (Non-Fe, closed diamonds) of magnetite particles are shown. Data are presented as the means of three independent cultures. Error bars represent standard deviations.

**Fig. 2:**
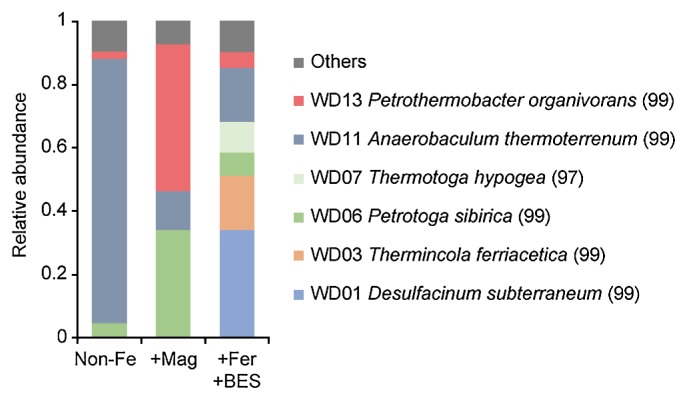
Phylogenetic distribution of bacterial 16S rRNA gene clones recovered from enrichment cultures supplemented with no additives (Non-Fe), magnetite (+Mag), and ferrihydrite plus BES (+Fer+BES). The dominant phylotypes (>5% in at least one enrichment) and their closest relatives (sequence identity, %) are shown in the legends.

**Fig. 3:**
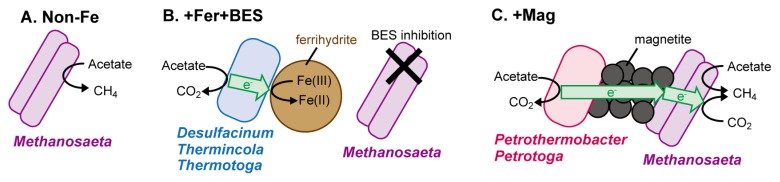
Schematic illustration of putative metabolic reactions, electron flows, and microbial species involved under each set of culture conditions. (A) Non-Fe, no iron added, (B) +Fer+BES, enriched with ferrihydrite and bromoethane sulfonate, and (C) +Mag, enriched with magnetite.
