# Supplementary material for: Conductive Iron Oxides Promote Methanogenic Acetate Degradation by Microbial Communities in a High-Temperature Petroleum Reservoir
Source: Microbes Environ. 2019 Feb 15;34(1):95–8. doi: 10.1264/jsme2.ME18140 (PMC6440731; doi:10.1264/jsme2.ME18140)
Supplement: Supplementary file 1 [file 34_95_s1.pdf]

Table S1. The bacterial phylotypes detected in the enrichment cultures.

Phylogenetic group	Phylotype	Closest relative (Similarity, %)	Number of clones		
			Non-Fe	+Mag	+Fer+BES
Deltaproteobacteria	WD01	AF385080 *Desulfacinum subterraneum* (99)			14
	WD02	AF385080 *Desulfacinum subterraneum* (89)			1
Firmicutes	WD03	NR_043042 *Thermincola ferriacetica* strain Z-0001 (99)			7
	WD04	NR_115129 *Desulfotomaculum kuznetsovii* strain 17 (84)			2
	WD05	EU652084 *Thermaerobacter subterraneus* strain mt-14 (89)	1		
Thermotogae	WD06	NR_025466 *Petrotoga sibirica* strain SL25 (99)	2	14	3
	WD07	AP014508 *Thermotoga hypogea* NBRC 106472 (97)			4
	WD08	DQ374393 *Thermotoga elfii* strain G1 (97)	2	2	
	WD09	NR_025389 *Thermosipho geolei* strain DSM 13256 (98)	1		
	WD10	NR_025466 *Petrotoga sibirica* strain SL25 (99)		1	
Synergistetes	WD11	NR_036784 *Anaerobaculum thermoterrenum* strain RWcit 16S (99)	35	5	7
	WD12	NR_074606 *Thermovirga lienii* strain DSM 17291 (89)			1
Deferribacteres	WD13	LC155975 *Petrothermobacter organivorans* strain ANA (99)	1	19	2
		Total clone numbers	42	41	41

Table S2. The archaeal phylotypes detected in the enrichment cultures.

Phylogenetic group	Phylotype	Closest relative (Similarity, %)	Number of clones		
			Non-Fe	+Mag	+Fer+BES
Euryarchaeota	WD14	NR_074214 *Methanosaeta thermophila* strain PT (100)	29	24	-*
		Total clone numbers	29	24	-

* Not analyzed because no PCR amplification was observed.
